# Dose-response meta-analysis of differences in means

**DOI:** 10.1186/s12874-016-0189-0

**Published:** 2016-08-02

**Authors:** Alessio Crippa, Nicola Orsini

**Affiliations:** Department of Public Health Sciences, Karolinska Institutet, Stockholm, Sweden

**Keywords:** Meta-analysis, Dose-response, Mean differences, Random-effects

## Abstract

**Background:**

Meta-analytical methods are frequently used to combine dose-response findings expressed in terms of relative risks. However, no methodology has been established when results are summarized in terms of differences in means of quantitative outcomes.

**Methods:**

We proposed a two-stage approach. A flexible dose-response model is estimated within each study (first stage) taking into account the covariance of the data points (mean differences, standardized mean differences). Parameters describing the study-specific curves are then combined using a multivariate random-effects model (second stage) to address heterogeneity across studies.

**Results:**

The method is fairly general and can accommodate a variety of parametric functions. Compared to traditional non-linear models (e.g. *E*_max_, logistic), spline models do not assume any pre-specified dose-response curve. Spline models allow inclusion of studies with a small number of dose levels, and almost any shape, even non monotonic ones, can be estimated using only two parameters. We illustrated the method using dose-response data arising from five clinical trials on an antipsychotic drug, aripiprazole, and improvement in symptoms in shizoaffective patients. Using the Positive and Negative Syndrome Scale (PANSS), pooled results indicated a non-linear association with the maximum change in mean PANSS score equal to 10.40 (95 % confidence interval 7.48, 13.30) observed for 19.32 mg/day of aripiprazole. No substantial change in PANSS score was observed above this value. An estimated dose of 10.43 mg/day was found to produce 80 % of the maximum predicted response.

**Conclusion:**

The described approach should be adopted to combine correlated differences in means of quantitative outcomes arising from multiple studies. Sensitivity analysis can be a useful tool to assess the robustness of the overall dose-response curve to different modelling strategies. A user-friendly R package has been developed to facilitate applications by practitioners.

## Background

The identification and characterization of dose-response relationships is an essential part of the analysis in many scientific disciplines such as toxicology, pharmacology, and epidemiology. This is particularly important in the development and testing of new compounds (e.g. a new drug, pharmaceutical treatment) where trials at different stages aim to evaluate the efficacy of increasing levels of dosage (Phase II-III trials) or to derive a dose-response curve for selection of optimal doses (Phase IV trials) [1, 2].

Randomized clinical trials often investigate a continuous outcome variable, such as the efficacy or safety of a drug, reporting the change from baseline of a medical score, or the final value of a clinical measurement. The dose-response results are typically summarized by dose-specific means and standard deviations [3]. Measures of effect are expressed in terms of mean or standardized mean differences using a dose level, usually the placebo group, as referent [1]. Over the last few years methodological research focused on developing and improving methods for performing dose-response analysis in a single study [4, 5]. A conclusive result is hardly obtained by a single investigation and there is often the need to synthesize information collected from multiple studies. In such a case meta-analytic methods can be used to define an overall relation or to investigate heterogeneity across study findings.

A method for pooling aggregated dose-response data where the outcome is a log relative risk was originally presented by Greenland and Longnecker in 1992 [6]. Since then, several papers have refined and covered specific aspects of the methodology such as model specification [7, 8], modeling strategies [9, 10], and software implementation [11, 12]. Other methodological articles extended the approach for continuous outcome but in the case where individual patient data are available, mainly in the context of time-series environmental studies [13–15].

Only a few alternatives have been proposed to pool aggregated dose-response data where the findings are summarized by differences in means. Davis and Chen [16] in 2004 described a methodology for summarizing dose-response curves of first and second generation antipsychotics in schizoaffective patients. The authors reconstructed drug-specific dose-response curves and conducted a meta-analysis to compare the effectiveness of medium vs high dosages. A common alternative to analyze the drug effect consists of fitting a random-intercept *E*_max_ model, where the random component accounts for heterogeneity in placebo effect across trials [17]. Heterogeneity, however, may be related to other study characteristics rather than differences in placebo response such as implementation, participants, intervention, and outcome definition. Thomas et al. [18] adopted hierarchical Bayesian models to summarize and describe, independently, the distribution of study-specific model parameters derived from an *E*_max_ model.

The mentioned strategies assumed pre-specified models that do not allow for non-monotonic curves which may occur in practice [19], as in case of dose-response data of antipsychotics. In addition, fitting study-specific sigmoidal curves such as the *E*_max_ model requires that the single studies have assessed at least three dose levels in order to estimate model parameters. Discarding studies not providing enough data points represents a loss of information and may introduce bias.

The aim of this paper is to formalize and propose a general and flexible methodology to pool dose-response relations from aggregated data where the changes in the distribution of the quantitative outcome are expressed in terms of differences in means. We first present the data necessary for a dose-response meta-analysis and derive formulas for obtaining effect sizes and their variance/covariance structure. We describe flexible dose-response models with particular emphasis on regression splines. The method is then applied to dose-response data from clinical trials on use of aripiprazole and symptoms improvement in schizoaffective patients.

## Methods

### Dose-response data

The notation and data required for a dose-response meta-analysis for a generic study are displayed in Table 1. We consider *I* studies indexed by *i*=1,…,*I* reporting the results of a common treatment at different dose levels *x*_*ij*_,*j*=1,…,*J*_*i*_, where *x*_0*i*_=0 indicates the control or placebo group in the *i*-th study. The study-specific results typically consist of dose-specific means of an outcome variable, *Y*_*ij*_, that measures the efficacy of the *j*-th dose in the *i*-th study [3]. Additional information about the number of patients allocated in each treatment, *n*_*ij*_, and the sample standard deviations of *Y*_*ij*_, *sd*_*ij*_, is generally reported or obtained from the study-specific results.

**Table 1:** Notation for aggregated data in the *i*-th study used in dose-response meta-analysis of differences in meas

dose	mean(Y)^*a*^	sd(Y)	*n* ^*b*^	d^*c*^	Var(d)	d^∗^^*d*^	Var(d^∗^)
0	$\bar Y_{i0}$	*s* *d*_*i*0_	*n*_*i*0_	0	–	0	–
⋮	⋮	⋮	⋮	⋮	⋮	⋮	⋮
*x*_*ij*_	$\bar Y_{ij}$	*s* *d*_*ij*_	*n*_*ij*_	*d*_*ij*_	Var(*d*_*ij*_)	$d^{*}_{ij}$	$\text {Var} \left (d^{*}_{ij} \right)$
⋮	⋮	⋮	⋮	⋮	⋮	⋮	⋮
$x_{iJ_{i}}$	$\bar Y_{iJ_{i}}$	$sd_{iJ_{i}}$	$n_{iJ_{i}}$	$d_{iJ_{i}}$	$\text {Var} \left (d_{iJ_{i}} \right)$	$d^{*}_{iJ_{i}}$	$\text {Var} \left (d^{*}_{iJ_{i}} \right)$

^a^Y is the continuous outcome

^b^Number of patients

^c^Mean difference

^d^Standardized mean difference

### Effect sizes and their variance/covariance

A common way to reduce heterogeneity in placebo response is to compute the effect size (or treatment effect) as difference between dose-specific means and placebo mean. In case all studies measure the outcome on a common and interpretable scale, the difference can be based on the absolute scale

1 $$ d_{ij} = \bar{Y}_{ij} - \bar{Y}_{i0}, \quad j = 1,\dots, J_{i}, \; i = 1, \dots, I   $$

Assuming common study-specific population standard deviations, the variance of *d*_*ij*_ is defined as

2 $$ \text{Var} \left(d_{ij} \right) = \frac{n_{ij}+ n_{i0}}{n_{ij} n_{i0}} s_{p_{i}}^{2}, \quad j = 1,\dots, J_{i}, \; i = 1, \dots, I   $$

where $s_{p_{i}}^{2} = \sum _{j = 0}^{J_{i}} \left (n_{ij} - 1 \right) sd_{ij}^{2} / \sum _{j = 0}^{J_{i}} \left (n_{ij} - 1 \right)$ is the square of the pooled standard deviation for the *i*-th study. Since the study-specific mean differences *d*_*ij*_ use the same referent values, $\bar {Y}_{i0}$, they cannot be regarded as independent. The covariance term is defined as

3 $$ \text{Cov} \left(d_{ij}, d_{ij^{'}} \right) = \text{Var} \left(\bar{Y}_{j0} \right) = \frac{s_{i0}^{2}}{n_{i0}}, \quad j \ne j^{'}, \; i = 1,\dots, I   $$

In case the outcome is measured on different scales the effect sizes can be based on standardized mean differences

4 $$ d_{ij}^{*} = \frac{\bar{Y}_{ij} - \bar{Y}_{i0}}{s_{p_{i}}}, \quad j = 1,\dots, J_{i}, \; i = 1, \dots, I   $$

with

5 $$\begin{array}{*{20}l}  \text{Var} \left(d_{ij}^{*} \right) &= \frac{1}{n_{ij}} + \frac{1}{n_{i0}} + \frac{{d_{ij}^{*}}^{2}}{2 \sum_{j=0}^{J_{i}} n_{ij}}, \quad j = 1,\dots, J_{i},\\ i &= 1, \dots, I  \\ \text{Cov} \left(d_{ij}^{*}, d_{ij^{'}}^{*} \right) &= \frac{1}{n_{i0}} + \frac{d_{ij}^{*}d_{ij^{'}}^{*}}{2 \sum_{j=0}^{J_{i}} n_{ij}}, \quad j \ne j^{'}, \; i = 1,\dots, I  \end{array} $$

### Dose-response analysis

The chosen effect sizes and the corresponding (co)variances are used to estimate the study-specific dose-response curves. The dose-response curves characterize the relative efficacy of the dose under investigation using the placebo effect as referent (i.e. the relative efficacy for the placebo is zero by definition). The dose-response models are expressed through the parametric model *f*, which specifies how the effect size varies according to the dose values. The functional relationship *f* is parametrized in terms of ***θ***_*i*_, the *p*×1 vector of dose-response coefficients. We consider the case of mean differences, *d*_*ij*_, but the same principles apply for standardized mean differences, $d^{*}_{ij}$. The study-specific curves can be written as

6 $$ \boldsymbol{d}_{i} = f\left(\boldsymbol{x}_{i}, \boldsymbol{\theta}_{i} \right) + \boldsymbol{\varepsilon}_{i}, \;\;\; \boldsymbol{\varepsilon}_{i} \sim N \left(\boldsymbol{0}, \boldsymbol{\hat \Sigma}_{i} \right), \quad i = 1, \dots, I   $$

$\boldsymbol {\hat \Sigma }_{i} $ is the covariance matrix of the residual error term, with Var(*d*_*ij*_) along the diagonal and $\text {Cov} \left (d_{ij}, d_{ij^{'}} \right)$ off-diagonal.

Several alternatives are available to model the dose-response curve (i.e. for the choice of *f*). Table 2 shows the definition of 4 types of models ordered according to the number of parameters, ranging from 1 for a linear model up to 3 for a logistic model. See Bretz et al. for a comprehensive description [2].

**Table 2:** Frequently used dose-response models

Model	Equation	No. of parameters
Linear	E[***d***_*i*_|***x***_*i*_]=*θ*_1*i*_ ***x***_*i*_	1
Quadratic	$\displaystyle \mathrm {E} \left [ \boldsymbol {d}_{i} | \boldsymbol {x}_{i} \right ] = \theta _{1i} \boldsymbol {x}_{i} + \theta _{2i} \boldsymbol {x}_{i}^{2}$	2
*E*_max_	$ \displaystyle \mathrm {E} \left [ \boldsymbol {d}_{i} | \boldsymbol {x}_{i} \right ] = \theta _{1i} \boldsymbol {x}_{i}^{\theta _{3i}}/ \left (\theta _{2i} + \boldsymbol {x}_{i}^{\theta _{3i}} \right)$	3
Logistic	E[***d***_*i*_|***x***_*i*_]=*θ*_1*i*_/{1+exp[(*θ*_2*i*_−***x***_*i*_)]/*θ*_3*i*_}	3

The most common choice in dose findings [2] is the use of the *E*_max_ model which is expressed in terms of three parameters: the maximum effect (*θ*_1*i*_), the dose to produce half of the maximum effect (*θ*_2*i*_) and the steepness of the curve (*θ*_3*i*_) [20]. As other non-linear models, the *E*_max_ model assumes a specific shape that does not allow for non-monotonic curve and its estimation requires at least three non reference dose levels. Quadratic models are defined by only *p*=2 coefficients but may poorly fit at extreme dose values [9]. Other non-linear models such as logistic and sigmoidal models, are commonly defined by *p*≥3 coefficients so that study-specific aggregated data may not be sufficient to estimate the parameters.

We propose the use of regression splines to flexibly model the dose of interest. Splines represent a family of smooth functions that can describe a wide range of curves (i.e. U-shaped, J-shaped, S-shaped, threshold) [21]. The curves consist of piecewise polynomials over consecutive intervals defined by ***k*** knots. Their use may facilitate curve fitting since many non-linear curves can be examined by estimating only a small number of coefficients. For instance, a restricted cubic spline model with three knots ***k***=(*k*_1_,*k*_2_,*k*_3_) is defined only in terms of *p*=2 coefficients [22]

7 $$ \mathrm{E} \left[ \boldsymbol{d}_{i} | \boldsymbol{x}_{i} \right] = \theta_{1i} \boldsymbol{x}_{1i} + \theta_{2i} \boldsymbol{x}_{2i}   $$

with two transformations [23] defined as

8 $$\begin{array}{*{20}l} x_{1} &= x  \\ x_{2} &= \frac{\left(x - k_{1} \right)_{+}^{3} - \frac{k_{3} - k_{1}}{k_{3} - k_{2}} \left(x - k_{2} \right)_{+}^{3} + \frac{k_{2} - k_{1}}{k_{3} - k_{2}} \left(x - k_{3} \right)_{+}^{3}}{ (k_{3} - k_{1})^{2}}  \end{array} $$

where the ‘+’ notation, with *u*_+_=*u* if *u*≥0 and *u*_+_=0 otherwise, has been used.

An alternative flexible approach to model the dose-response association is represented by fractional polynomials. In particular, a dose-response model based on fractional polynomial of order two can be written as in Eq. 6 with the two transformations defined as

9 $$\begin{array}{*{20}l} x_{1} &= x^{p_{1}} \text{ and } x_{2} = x^{p_{2}} \text{ if \(p_{1} \neq p_{2}\)}  \\ x_{1} &= x^{p_{1}} \text{ and } x_{2} = {x}^{p_{1}} \log({x}) \text{ if \(p_{1} = p_{2}\) }  \end{array} $$

for each combination of *p*_1_ and *p*_2_ in the predefined set of values {−2,−1,−0.5,0,0.5,1,2,3}; for *p*=0, *x*^*p*^ becomes log(*x*). The best fitting fractional polynomial is typically chosen based on the Akaike’s Information Criterion [24].

Once the functional relation *f* has been selected, generalized least square estimation can be performed to efficiently estimates the dose-response coefficients $\boldsymbol {\hat \theta }_{j}$ and the corresponding (co)variance matrix $\boldsymbol {\hat V}_{j}$, by minimizing

10 $$ \left(\boldsymbol{d}_{i} - f\left(\boldsymbol{x}_{i}, \boldsymbol{\theta}_{i} \right) \right)^{T} \boldsymbol{\hat \Sigma}_{i}^{-1} \left(\boldsymbol{d}_{i} - f\left(\boldsymbol{x}_{i}, \boldsymbol{\theta}_{i} \right) \right)   $$

that generally requires numerical optimization algorithms. If *f* is a linear combination of the parameters ***θ***_*i*_, as in the case of regression splines and fractional polynomials, the close solution can be written as

11 $$\begin{array}{*{20}l} \boldsymbol{\hat \theta}_{i} &= \left(\boldsymbol{X}_{i}^{T} \boldsymbol{\hat \Sigma}_{i}^{-1} \boldsymbol{X}_{i} \right)^{-1} \boldsymbol{X}_{i}^{T} \boldsymbol{\hat \Sigma}_{i}^{-1} \boldsymbol{d}_{i}  \\ \boldsymbol{\hat V}_{i} &= \text{Var} \left(\boldsymbol{\hat \theta}_{i} \right) = \left(\boldsymbol{X}_{i}^{T} \boldsymbol{\hat \Sigma}_{i}^{-1} \boldsymbol{X}_{i} \right)^{-1}  \end{array} $$

where ***X***_*i*_ indicates the *J*_*i*_×*p* design matrix in the *i*-th study.

### Meta-analysis

The estimated study-specific dose-response coefficients $\boldsymbol {\hat \theta }_{i}$ and the accompanying (co)variance matrices $\boldsymbol {\hat V}_{i}$ are combined by means of multivariate meta-analysis

12 $$ \boldsymbol{\hat \theta}_{i} \sim N \left(\boldsymbol{\theta}, \boldsymbol{\hat V}_{i} + \boldsymbol{\Psi} \right)   $$

A fixed-effects model assumes no statistical heterogeneity among study results, i.e. differences in the dose-response coefficients are only related to sampling error. The assumption of homogeneity may not hold in practice, unless it is known that the studies are performed in a similar way and are sampled from the same population [25]. The Cochran’s Q test [26] is typically used to test statistical heterogeneity across studies (*H*_0_:***Ψ***=***0***) [27]. Selected studies, however, will typically differ with respect to study design and implementation, selection of participants, and type of analyses. A certain degree of heterogeneity is expected and should be taken into account in the analysis. A random-effects model allows the dose-response coefficients, ***θ***_*i*_, to vary across studies. Statistical heterogeneity is captured by the between-studies variance ***Ψ*** while ***θ*** represents the mean of the distribution of dose-response coefficients and an estimate, $\boldsymbol {\hat \theta }$, can be obtained using (restricted) maximum likelihood estimation [15].

As a final result, the pooled dose-response curve can be presented in either a graphical or tabular form by predicting the mean differences of the outcome for a set of *x* dose values

13 $$ \mathrm{E} [ \boldsymbol{\hat{d}} | \boldsymbol{x} ] = f\left(\boldsymbol{X}, \boldsymbol{\hat \theta} \right)   $$

with an approximate (1−*α*/2)% confidence interval (CI), that in case *f* is a linear combination of ***θ*** can be expressed as

14 $$ \mathrm{E} [ \boldsymbol{\hat{d}} | \boldsymbol{x} ] \mp z_{\frac{\alpha}{2}} \sqrt{\text{diag} \left(f\left(\boldsymbol{X}, \boldsymbol{\hat \theta} \right)^{T} \text{Cov}\left(\boldsymbol{\hat \theta} \right) f\left(\boldsymbol{X}, \boldsymbol{\hat \theta} \right) \right)}   $$

where *z*_*α*/2_ is the *α*/2-th quantile of a standard normal distribution.

### Dose findings

Once the pooled dose-response curve has been estimated, it may be of interest to determine a set of target doses, i.e. doses associated with prespecified outcome effects. In development of new compounds it is often important to select an optimal dose which is almost as effective as the maximum effective dose but has less undesired side effects, which often occur at high dosages. Suppose one wants to determine which is the lowest dose (ED_*γ*_) to produce an almost complete effect, e.g. *γ%* of the observed maximum predicted response.

The ED_*γ*_ can be determined as

15 $$ \mathrm{\widehat{ED}}_{\gamma} = \underset{x \in \left(0, x_{\text{max}} \right]}{\operatorname{argmax}} \left\{ \frac{\mathrm{E} [ {\hat{d}} | {x} ] }{\mathrm{E} [ {\hat{d}} | {{x_{\text{max}}}} ]} \geq \gamma \right\}   $$

where *x*_max_ is the dose corresponding to the maximum predicted outcome.

An important step when presenting results from dose findings analysis is to accompany the previous estimates with a measure of precision, typically confidence intervals. Pinheiro et al. [3] proposed the use a parametric bootstrap approach based on the asymptotic normal distribution of $\boldsymbol {\hat \theta }$, the pooled estimate of the dose-response coefficients. The approach consists in re-sampling the dose-response coefficients ***θ*** from its approximate normal distribution and derive the distribution of $\mathrm {\widehat {ED}}_{\gamma }$ based on the samples. Approximated confidence intervals for $\mathrm {\widehat {ED}}_{\gamma }$ can be constructed using percentiles of the sampling distribution.

## Results

To illustrate the methodology we examined the dose-response relation between aripiprazole, a second-generation antipsychotic, and symptoms improvement in schizoaffective patients. We updated the search strategy presented in a previous review by Davis and Chen [16] by searching the Medline, International Pharmaceutical Abstracts, CINAHL, and the Cochrane Database of Systematic Reviews. To reduce the exclusion of unpublished papers, additional sources including Food and Drug administration website, data from Cochrane reviews, poster presentations and conference abstracts were also searched. All random-assignment, double-blind, controlled clinical trials of schizoaffective patients providing dose-response results for at least two non-zero dosages of aripiprazole were eligible.

Five studies [28–32] met the inclusion criteria and were included in the analysis. All the studies reported mean changes from baseline as main outcome variable, using the Positive and Negative Syndrome Scale (PANSS). The PANSS scale is an ordinal score derived from 30 items ranging from 1 to 7. Computations of ratios such as percentage changes are not directly applicable and may lead to erroneous results [33, 34]. To address this issue, the theoretical minimum (i.e. 30) needs to be subtracted from the original score [35]. Information about the means, the number of patients assigned to each treatment, and the standard deviation was available from the published data. Because all the studies measured the outcome variable on the same scale, we computed PANSS mean differences as effect sizes. Data are reported in Table 3.

**Table 3:** Aggregated dose-response data of five clinical trials investigating effectiveness of different dosages of aripiprazole in schizoaffective patients. The continuous outcome is measured on the Positive and Negative Syndrome Scale and summarized by mean values (mean(Y)) and standard deviations (sd(Y))

ID	Author, Year	dose	mean(Y)	sd(Y)	n	d	Var(d)
1	Cutler, 2006 [28]	0	5.300	18.310	85	0.000	0.000
		2	8.230	18.320	92	2.930	7.593
		5	10.600	18.310	89	5.300	7.715
		10	11.300	18.320	94	6.000	7.515
2	McEvoy, 2007 [29]	0	2.330	26.100	107	0.000	0.000
		10	15.040	27.600	103	12.710	13.344
		15	11.730	26.200	103	9.400	13.344
		20	14.440	25.900	97	12.110	13.764
3	Kane, 2002 [30]	0	2.900	24.280	102	0.000	0.000
		15	15.500	26.490	99	12.600	12.038
		30	11.400	22.900	100	8.500	11.977
4	Potkin, 2003 [31]	0	5.000	21.140	103	0.000	0.000
		20	14.500	20.160	98	9.500	8.563
		30	13.900	20.880	96	8.900	8.654
5	Study 94202 [32]	0	1.400	25.730	57	0.000	0.000
		2	11.000	25.000	51	9.600	25.447
		10	11.500	25.200	51	10.100	25.447
		30	15.800	28.510	54	14.400	24.701

We used the trial by Cutler et al. [28] to illustrate the steps required for estimating the dose-response curve for a single study. For example, the difference in mean PANSS comparing the dose of 2 mg/day relative to 0 mg/day is *d*_11_=8.23−5.3=2.93 mg/day. Its variance is $\text {Var}(d_{11}) = (85+92)/(85\times 92)\times s_{p_{1}}^{2}$ = 7.59, where $s_{p_{1}}^{2}=119,419/356=335.4$. The covariance of this difference in means PANSS is 18.31^2^/85=3.94. The variance/covariance structure associated with the vector of differences in means for this trial ***d***_***1***_ can be presented in a matrix form

$$\begin{array}{@{}rcl@{}} \boldsymbol{\hat \Sigma_{1}} =\left[ \begin{array}{lll} 7.59 & & \\ 3.94 & 7.72 & \\ 3.94 & 3.94 & 7.52 \\ \end{array}\right] \end{array} $$

To estimate the dose-response curve we need first to specify the model *f*. We characterized the dose-response relation using a restricted cubic spline model with three knots located at the 10th, 50th, and 90th percentiles (0, 10, and 30 mg/day) of the overall dose distribution (*p* = 2). The restricted cubic spline dose-response model is defined as in Eq. 7. Efficient estimates of the dose-response coefficients and (co)variance matrix were obtained by generalized least square estimation

16 $$\begin{array}{*{20}l} \boldsymbol{\hat \theta}_{1} &= (1.215, -5.738)^{T}  \\ \boldsymbol{\hat V}_{1} &= \text{Var} \left(\boldsymbol{\hat \theta}_{1} \right) =\left[ \begin{array}{ll} 0.49 & \\ -3.65 & 31.64 \\ \end{array}\right] \end{array} $$

**Fig. 1:**
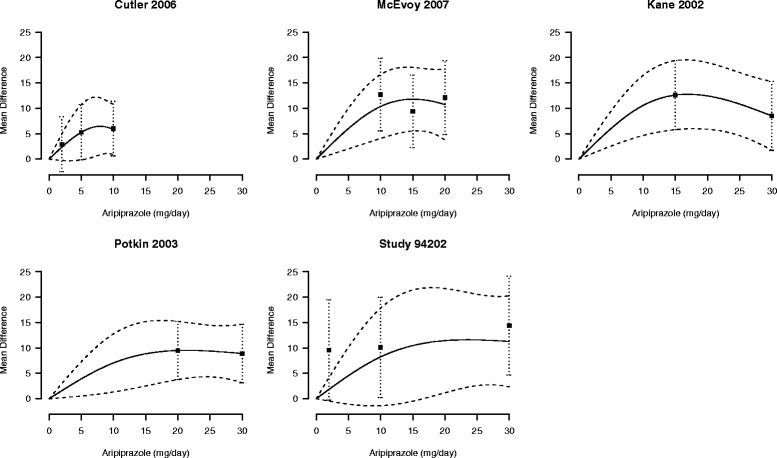
Study-specific mean differences in Positive and Negative Syndrome Scale score for increasing dosages of aripiprazole. The first author and year of publication of the subjects included in the original analyses are reported. Black squares indicate the mean differences and whiskers their 95 % confidence interval. Ariprazole dosage was modeled with restricted cubic splines. Solid lines represent the estimated dose-response curves, dashed lines the corresponding 95 % confidence intervals. The placebo group (dose = 0) served as the referent group

We applied the same procedure to the other studies included in the meta-analysis in order to obtain the study-specific $\boldsymbol {\hat \theta }_{i}$ and $\boldsymbol {\hat {V}}_{i}$, *i*=1,…,5 (Table 4). The study-specific predicted curves are presented in Fig. 1. Under a random-effects model, restricted maximum likelihood estimates were

17 $$ \begin{aligned} \boldsymbol{\hat \theta} &= (0.937, -1.156)^{T} \\ \widehat{\text{Cov}} \left(\boldsymbol{\hat \theta} \right) &= \left[\begin{array}{cc} 0.03 & \\ -0.05 & 0.10 \\ \end{array}\right] \end{aligned}  $$

**Table 4:** Study-specific dose-response coefficients and corresponding covariances for different dose-response models considered in the analysis

Model	id	$\hat \theta _{1}$	$\hat \theta _{2}$	$\text {Var} \left (\hat \theta _{1} \right)$	$\text {Cov} \left (\hat \theta _{1},\hat \theta _{2} \right)$	$\text {Var} \left (\hat \theta _{2} \right)$
Restricted cubic splines	1	0.55	0.55	0.065	0.065	0.065
	2	0.59	0.59	0.031	0.031	0.031
	3	0.84	−1.69	0.054	−0.12	0.38
	4	0.47	−0.54	0.021	−0.043	0.15
	5	0.78	−1.25	0.23	−0.62	1.8
Fractional Polynomials	1	17.47	−7.84	2.9e+02	−2.2e+02	1.8e+02
	2	29.59	−12.42	2.5e+02	−1.6e+02	1e+02
	3	32.12	−13.47	1.4e+02	−70	37
	4	18.48	−6.42	1.8e+02	−93	49
	5	21.97	−7.00	2.3e+02	−1.1e+02	55
Emax	1	8.13	3.13	27	24	36
	2	11.39	0.00	38	35	42
	3	10.54	0.00	37	53	1e+02
	4	9.20	0.00	60	1.4e+02	3.6e+02
	5	13.28	0.94	23	2.7	2.7
Quadratic	1	1.54	−0.09	0.96	−0.086	0.0083
	2	1.54	−0.05	0.35	−0.017	0.00089
	3	1.40	−0.04	0.18	−0.0055	0.00018
	4	0.83	−0.02	0.14	−0.0047	0.00017
	5	1.08	−0.02	0.51	−0.016	0.00051
Piecewise linear	1	0.55		0.065		
	2	0.59		0.031		
	3	0.84	−1.69	0.054	−0.12	0.38
	4	0.47	−0.54	0.021	−0.043	0.15
	5	0.78	−1.25	0.23	−0.62	1.8

A *p*-value < 0.001 for the multivariate Wald-type test *H*_0_:***θ***=***0*** provided strong evidence against the null hypothesis of no relation between different doses of aripiprazole and mean change PANSS score. The Q test (*Q*=3.5, *p*-value = 0.899) did not detect substantial statistical heterogeneity across studies.

**Fig. 2:**
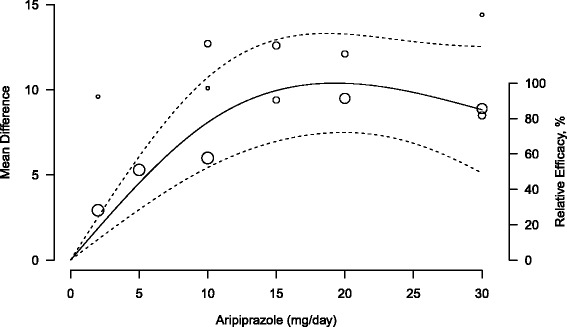
Pooled dose-response association between aripiprazole and mean change in Positive and Negative Syndrome Scale score (*solid line*). Aripiprazole dosage was modeled with restricted cubic splines in a random-effects model. Dash lines represent the 95 % confidence intervals for the spline model. The placebo group (dose = 0) served as the referent group. Circles indicate observed mean differences in individual studies; size of bubbles is proportional to precision (inverse of variance) of the mean differences. Right axis represents percentage of the maximum predicted effect

To communicate results of the pooled dose-response analysis, we can estimate the pooled mean differences in PANSS scores using 0 mg/day as referent as 0.937*x*_1_−1.156*x*_2_, together with the corresponding 95 % confidence interval for a generic dose *x* of interest as following

$$\left(0.937 x_{1} -1.156 x_{2} \right) \mp 1.96 \sqrt{0.03 {x_{1}^{2}} + 0.1 {x_{2}^{2}} - 0.1x_{1}x_{2}} $$

where *x*_1_ and *x*_2_ are defined as in Eq. 8. For instance, the model-based predicted mean changes in PANSS score compared to placebo were 4.52 (95 % CI: 2.96, 6.08) for 5 mg/day, 8.08 (95 % CI: 5.43, 10.73) for 10 mg/day, 9.95 (95 % CI: 6.97, 12.94) for 15 mg/day, 10.38 (95 % CI: 7.49, 13.27) for 20 mg/day, 9.84 (95 % CI: 6.86, 12.83) for 25 mg/day, and 8.83 (95 % CI: 5.11, 12.54) for 30 mg/day. The pooled predicted dose-response curve together with the confidence intervals and the model mean differences is provided in Fig. 2.

The results indicated a statistically significant positive association between increasing doses of aripiprazole and the mean change in PANSS score with the maximum value of 10.39 (95 % CI: 7.48, 13.30) observed at *x*_max_ = 19.32 mg/day. The model suggested a slight decrease in the predicted mean PANSS score for dosages greater than 20 mg/day. The estimated dose to produce 50 % and 80 % of the predicted maximum effect were $\mathrm {\widehat {ED}}_{50} =$ 5.82 mg/day (95 % CI: 5.10, 8.58) and $\mathrm {\widehat {ED}}_{80} =$ 10.43 mg/day (95 % CI: 9.02, 16.73).

### Sensitivity analysis

**Fig. 3:**
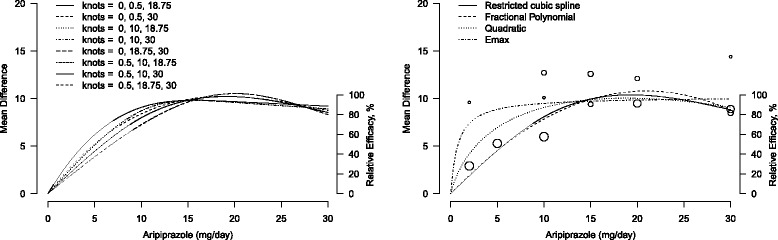
Graphical sensitivity analysis for the pooled dose-response curves between aripiprazole and mean change in Positive and Negative Syndrome Scale score. The placebo group (dose = 0) served as the referent group. Right axis represents percentage of the maximum predicted effect. Left panel: different location of the three knots in a restricted cubic spline model. Right panel: different models, restricted cubic splines (*solid line*), fractional polynomials (*dashed line*), quadratic polynomial (*dotted line*), and *E*_max_ model (*dot-dashed line*). Circles indicate observed mean differences in individual studies; size of bubbles is proportional to precision (inverse of variance) of the mean differences

A sensitivity analysis is often required to evaluate the robustness of the pooled dose-response curve. In the spline model, for example, the location of the knots may affect the shape of the dose-response curve. Therefore we considered alternative knots locations including different combinations of the 10th, 25th, 50th, 75th and 90th percentiles of the overall dose distribution (0, 0.5, 10, 18.75, and 30 mg/day). A graphical comparison is presented in the left panel of Fig. 3. The alternative curves roughly described the same dose-response shape with no substantial variation, all indicating an increase in the mean change PANSS score up to 20 mg/day of aripiprazole. We can assess whether there is an increasing trend above 20 mg/day by simply re-defining *x*_2_ equal to (*x*−20)_+_ in Eq. 8; this approach is known as piecewise linear model. The rate of change in the PANSS mean differences was negative and not statistically significant (*θ*_1_+*θ*_2_=-0.284, *p* = 0.18) after 20 mg/day of aripiprazole.

To evaluate the sensitivity of the dose-response curve to the choice of the parametric model *f* adopted, instead, we considered three alternatives: fractional polynomials; quadratic; and *E*_max_. Since two studies only had two non-referent doses, the study-specific (sigmoidal) *E*_max_ models as described in Table 2 cannot be estimated. A common solution is to fix the steepness of the curve *θ*_3_ to be 1, also referred to as hyperbolic *E*_max_ [20].

The “best” fractional polynomials (*p*_1_=0.5, *p*_2_=1) provided overall a similar dose-response curve when compared to the spline model, with slightly higher value for the maximum predicted response (right panel of Fig. 3). The hyperbolic *E*_max_ had substantially higher predicted mean differences for low values of the dose. The non-linear model assumes a specific hyperbolic dose-response curve that did not seem to fit the data and may be dependent from the choice of fixing *θ*_3_ to be 1. The dose-response curve described by the quadratic model fall in between the spline and the hyperbolic *E*_max_ curves.

## Discussion

In this paper we proposed a statistical method to combine differences in means of quantitative outcomes. The method consists of dose-response models estimated within each study (first stage) and an overall curve obtained by pooling study-specific dose-response coefficients (second stage). The covariance among study-specific mean differences is taken into account in the first stage analysis using generalized least square estimators, while statistical heterogeneity across studies is allowed by multivariate random-effects model in the second stage.

One major strength of the proposed method is that it is fairly general and can accommodate different modeling strategies, including non-linear ones described by Pinheiro et al. [3]. Non-linear models, however, are defined by at least three or four parameters, and hence require an equal number of dose levels for each single study included in the analysis. Given that some studies may have investigated a lower number of dose levels, exclusion of these studies may result in substantial loss of information. In addition, many non-linear models assume a specific behaviour (e.g. monotonicity) requiring a strong a priori information about the dose-response curve. The choice of the parametric model is critical, since it highly influences the final results [3]. Indeed, the selection of the dose-response model should be informed by subject-matter knowledge as well as understanding of the research questions at hand. We presented the use of regression splines as a flexible tool for modeling any quantitative exposure. The major advantage is that a variety of curves, even non monotonic ones, can be estimated using only two parameters. It is considered to be closed to non-parametric regression, since no major assumptions about the shape of the curve are needed [9]. A possible alternative is the use of fractional polynomials. In comparing the two strategies, we did not find important differences between the two strategies and concluded that both are useful tools to characterize a (non-linear) dose-response curve. Nonetheless a sensitivity analysis is generally required to evaluate the robustness of the combined results.

A possible limitation of the proposed methodology is that it requires information about dose-specific means and standard deviations. Studies providing other summary measures, such as dose-specific medians, would not be included the analysis. The dose-response analysis presented in Eq. 6 is based on the asymptotic normal distribution of the conditional mean effect size. Extension of the introduced methodology to percentiles is not straightforward and may represent an interesting topic of future research.

An additional limitation of aggregated dose-response data is that supplementary information for approximating the covariance terms may not be available. Articles may report directly mean or standardized mean differences and standard errors for non-referent dose groups. Whenever the standard deviation for the outcome variable in the control group (${s_{i0}^{2}}$) cannot be obtained, it may be approximated using the pooled standard deviation based on the non-referent dose levels ($s_{p_{ij}}^{2}$). Alternatively a specific value may be imputed and a sensitivity analysis can be performed to evaluate how the results of the meta-analysis vary for different values of ${s_{0j}^{2}}$. Further limitations relate to the general application of meta-analysis based on aggregated data. These include restrictions in subgroup analyses, the impossibility of assessing the appropriateness of individual analyses, and to harmonize variable definitions and analyses for reducing the extent of heterogeneity, as well as specific biases such as aggregation (or ecological) bias in meta-regression models. Meta-analysis of individual patient data, however, are often difficult to undertake especially for the availability of individual data, so that usage of aggregated data may represent the only alternative [36]. Specific to aggregated dose-response data, different dose references and exposure range may complicate the analysis. The presented methodology assume that all the selected studies share a common dose-response model. Important departure from this assumption may limit and/or impact the pooling of individual dose-response coefficients. An alternative methods has been proposed based on a series of univariate meta-analyses of effect sizes for a pre-specified grid of dose-levels [37]. Further work is needed to analyze this possibility and potential advantages. Depending on the extent of heterogeneity of the dose-response curves, however, it may not be opportune to pool study-specific results, and meta-regression or stratified analyses should be performed [38].

In our application, we considered the effectiveness of increasing dosages of aripiprazole in shizoaffective patients. We described the steps needed to obtain the overall dose-response curve and to present it in a graphical form. We observed a non-linear association with the maximum efficacy corresponding to aripiprazole 19.32 mg/day. An estimated dose of 10.46 mg/day, however, may be sufficient to obtain 80 % of the maximum effect, which may be relevant for avoiding possible undesired side effects. Sensitivity analysis showed similar results as compared to fractional polynomials. The *E*_max_ model presented higher drug efficacy for low dosages. Compared to the previous models, the *E*_max_ model did not seem to fit properly the data at low dosages.

## Conclusions

We described an approach to combine differences in means of a quantitative outcome contrasting different dose levels relative to a placebo in randomized trials. The general framework of the proposed methodology can include a variety of flexible models. Sensitivity analysis can be a useful tool to assess the stability of the overall dose-response curve to different modelling strategies. Although the method was presented for the analysis of randomized trials, it may be extended to observational studies where mean differences are further adjusted for potential confounders. Future work is needed to evaluate the properties of the statistical model and validity of the underlying assumptions. A user friendly procedure is implemented in the dosresmeta R package [39] with worked examples available on GitHub.

## Abbreviations

PANSS, positive and negative syndrome scale
